# Comparison of Enoxaparin and Rivaroxaban in the Prophylaxis of Deep Venous Thrombosis in Arthroplasty

**DOI:** 10.1155/2021/2945978

**Published:** 2021-11-16

**Authors:** Necati Çiçek, İsmail Ağir, Hacı Bayram Tosun, Abuzer Uludağ, Abdulkadir Sari

**Affiliations:** ^1^Department of Orthopedics and Traumatology, Adıyaman Training and Research Hospital, Adıyaman, Turkey; ^2^Department of Orthopedics and Traumatology, Adiyaman University, School of Medicine, Adıyaman, Turkey; ^3^Department of Orthopedics and Traumatology, Taksim Training and Research Hospital, Istanbul, Turkey; ^4^Department of Orthopedics and Traumatology, Namık Kemal University, School of Medicine, Tekirdağ, Turkey

## Abstract

**Background:**

Pulmonary embolism is a serious early complication of arthroplasty procedures that can develop after deep venous thrombosis. The present study aimed to compare rivaroxaban and enoxaparin in terms of preventing DV and PE, and also in this study, we compared the complications due to these drugs in patients undergoing elective arthroplasty.

**Materials and Methods:**

214 patients were divided into three groups based on their treatment regimens. In group I, enoxaparin was used, in group II, rivaroxaban was used, and in group III, enoxaparin was used throughout hospitalization, and after hospital discharge, rivaroxaban was used. These three groups were compared according to the occurrence of deep venous thrombosis, pulmonary embolism, and major and minor complications.

**Results:**

Major postoperative complications occurred in 5, 15, and 6 patients in group I, II, and III, respectively. Minor postoperative complications occurred in 10, 24, and 11 patients in group I, II, and III, respectively. No significant difference was found among the three groups. Deep venous thrombosis or pulmonary embolism was not observed in any patient.

**Conclusion:**

Rivaroxaban was found to be as effective as enoxaparin in the prevention of deep venous thrombosis and other complications after arthroplasty. Moreover, oral rivaroxaban provided greater ease of use compared to subcutaneous enoxaparin. Based on these findings, we consider that rivaroxaban could be an effective alternative to enoxaparin.

## 1. Introduction

Deep venous thrombosis (DVT) is a prevalent clinical condition most commonly seen in deep veins of lower extremities and rarely seen in upper extremities, pelvis, and other veins. Pulmonary embolism (PE) is the most important complication of DVT which threatens the life [1]. DVT is a major complication in total knee arthroplasty (TKA) and total hip arthroplasty (THA), leading to increased postoperative mortality and morbidity [2]. Moreover, DVT may show various manifestations ranging from asymptomatic deep venous thrombosis (DVT) to fatal PE [3]. Symptomatic or asymptomatic DVT can be seen in 50% of patients undergoing THA without thromboprophylaxis and in up to 60% of patients undergoing TKA (1, 3). Additionally, symptomatic findings of DVT may be seen in approximately 15–30% of patients undergoing THA and TKA [4, 5].

For these reasons, prophylactic treatment is of prime importance for reducing the risk of DVT and morbidity in patients undergoing lower extremity arthroplasty. In patients undergoing TKA, the incidence of DVT is remarkably high, and the incidence of DVT is higher than that of THA [4, 6, 7]. Nevertheless, the incidence of venous thromboembolism (VTE) has decreased over time with the advancements in surgical techniques and VTE prevention methods [4]. Of particular importance, the use of VTE prophylaxis had reduced the incidence of symptomatic DVT to approximately 1–3% and the incidence of PE to approximately 0.2–1.1% [3, 8–11].

Low-molecular-weight heparins (LMWH) are anticoagulants which are used frequently, and they inhibit the coagulation process through binding to antithrombin via a pentasaccharide sequence. Rivaroxaban is the other anticoagulant which inhibits both free factor Xa and factor Xa bound in the https://en.wikipedia.org/wiki/Prothrombinase and has oral use. Protamine sulfate is a medication that is used to reverse the effects of LMWH. Until the last years, there are no antidotes available for rivaroxaban which is a problem in the case of overdose, major bleeding, or urgent surgical intervention [12]. Therefore, its use by orthopedic surgeons was viewed with suspicion, but when we look at the literature, new products used as antidotes have been produced in recent years. One of them is andexanet alfa which is the first US Food and Drug Administration (FDA) approved agent for the reversal of anticoagulation in patients treated with apixaban or rivaroxaban. Andexanet alfa's procoagulant effects are achieved through the ability to bind and sequester factor Xa inhibitors [13].

The present study was designed to investigate the efficacy of prophylactic use of LMWH (enoxaparin) and rivaroxaban in terms of preventing DVT and PE, and also in this study, we compared the complications due to these drugs in patients undergoing elective arthroplasty.

## 2. Materials and Methods

This retrospective study included 214 patients between January 2017 and August 2019. The study was approved by the Adiyaman University local ethics committee (approval no. 19-6.1T/49), and informed consent was obtained from each patient.

Patients were divided into three groups based on their treatment regimens:

- In group I, the first dose of enoxaparin (Clexane 4000 anti-Xa IU/0.4 ml) was administered subcutaneously at postoperative 12 h (after bleeding control), and the treatment was continued for a total period of 15 days.
- In group II, the first dose of rivaroxaban (Xarelto^®^ 10 mg) was administered perorally at postoperative 12 h, and the treatment was continued for a total period of 15 days.
- In group III (combined group), the first dose of enoxaparin was administered subcutaneously at postoperative 12 h, and the treatment was continued throughout hospitalization. Following hospital discharge, the patient received rivaroxaban 10 mg perorally for a total period of 15 days.

The 214 patients comprised 166 (77.6%) women and 48 (22.4%) men. Of all patients, 178 (83.2%) underwent TKA, and 36 (16.8%) underwent THA. Mean age was 65 years (range: 25–89 years), with a mean age of 62 years (range: 27–82 years) in group I, 64 years (range: 25–79 years) in group II, and 67 years (range: 28–89 years) in group III. Mean operative time was 101.98 min (range: 90–135 min), with a mean operative time of 99.13 min (range: 90–135 min) in group I, 102.4 min (range: 90–135 min) in group II, and 103.5 min (range: 90–130 min) in group III. No significant difference was found among the three groups with regard to operative time (*p* > 0.001).

The inclusion criteria were as follows:

- Primary or secondary coxarthrosis
- Gonarthrosis

The exclusion criteria were as follows:

- Prior VTE or DVT
- Use of antithrombotic or anticoagulant drugs prior to the study
- History of major orthopedic surgery within the last one month
- History of brain or eye surgery within the last three months
- Prior malignancy
- History of myocardial infarction or stroke within the last six months
- Allergy to heparin or iodine-radiopaque material
- Antithrombin, protein C, or protein S deficiency
- Severe uncontrollable hypertension (HT) (diastolic blood pressure >120 mmHg; systolic blood pressure >200 mmHg)
- Hepatic and renal dysfunction

The uncemented implants were used in patients undergoing THA, and the cemented implants were used in patients undergoing TKA. Spinal anesthesia was used for these procedures. Tourniquet and hemovac drain was used in TKA, and the tourniquet was released after skin closure. Hemovac drain was removed on the second postoperative day. In all patients, an elastic bandage or compression stocking was applied throughout two weeks, and the patients were mobilized within 4 days after surgery.

Major postoperative complications were defined as fatal bleeding, blood loss leading to a decrease of more than 2 g/dl in hemoglobin (Hb), blood loss leading to more than two units of blood transfusion, blood loss leading to discontinuation of treatment or requiring reoperation, and symptomatic retroperitoneal, intracranial, intraocular, or intraspinal bleeding. Minor postoperative complications were defined as ecchymosis with an area of more than 25 cm^2^, wound site hematoma, spontaneous nasal or gingival bleeding lasting longer than five minutes, spontaneous rectal bleeding, spontaneous macroscopic hematuria, or hematuria lasting longer than 24 hours in the presence of urinary catheters. Both major and minor postoperative complications were recorded and compared among the three groups using statistical analyses.

### 2.1. Statistical Analysis

Data were analyzed using SPSS for Windows version 22.0 (Armonk, NY: BMI Corp.). Descriptives were expressed as mean ± standard deviation (SD). Complication rates were determined using the chi-square test and Cramer's V, and continuous data were compared using the one-way ANOVA tests. A *p* value <0.05 was considered significant.

## 3. Results

DVT or PE was not observed clinically in any patient in all three groups according to clinical examination and symptoms.

Mean duration of postoperative hospital stay was 5.4 days (range: 2–14 days), with a duration of 5.13 days (range: 3–11 days) in group I, 5.21 days (range: 2–9 days) in group II, and 6.1 days (range: 3–14 days) in group III, respectively. No significant difference was found among the three groups with regard to the duration of postoperative hospital stay (*p*=1.00).

Major postoperative complications occurred in 5 (10.9%), 15 (12.2%), and 6 (13.3%) patients in group I, II, and III, respectively. Blood loss leading to a decrease of more than 2 g/dl in Hb was observed in all 5 patients in group I and in 5 patients in group II, while blood loss leading to more than two units of blood transfusion was observed in 10 patients in group II and in all 6 patients in group III. No significant difference was found among the three groups with regard to major postoperative complications (*p*=0.974) (Table 1).

Minor postoperative complications occurred in 10 (22.2%), 24 (19.5%), and 11 (24.4%) patients in group I, II, and III, respectively. No significant difference was found among the three groups with regard to minor postoperative complications (*p*=0.779) (Table 2).

## 4. Discussion

Deep venous thrombosis is a frequent complication seen in THA and TKA, leading to high mortality and morbidity due to surgical procedures [14].

Many agents are used for prophylaxis after arthroplasty because of the risk of DVT. Many novel investigations have been conducted to find the most effective anticoagulants in asymptomatic DVT prophylaxis due to many factors including the differences in the mechanism of action of these agents in the coagulation cascade in reaching the drug plasma concentrations, the need for monitoring during the administration of these agents, and the side effects of these drugs. Bauersachs et al. [15] suggested that rivaroxaban alone is as efficacious as standard therapy, with similar safety, for the treatment of acute DVT, and when treatment is continued, rivaroxaban is highly effective in preventing recurrences, as compared with placebo, and also has an acceptable risk of bleeding. Similarly, in our study, rivaroxaban alone was as effective as LMWH in preventing DVT. Additionally, the same study conducted by Bauersachs et al. also noted that rivaroxaban could be used as a single agent instead of both LMWH and a vitamin K antagonist in the prophylaxis of DVT [15]. In our study, rivaroxaban alone was administered in group II for DVT prophylaxis, and similar outcomes were obtained. Additionally, the efficacy of the treatments administered in all three groups was similar during the first several weeks. This study shows that rivaroxaban alone might further facilitate the outpatients in prophylaxis of DVT [16–19].

In a meta-analysis study, Suen et al. [20] reviewed 45 randomized controlled studies including a total of 56,700 patients who have been applied prophylaxis with more and/or one drugs such as LMWH, rivaroxaban, warfarin, apixaban, aspirin, and dabigatran or no pharmacologic treatment in terms of the risk of surgical site bleeding after TKA or THA. The authors indicated that the bleeding risk in LMWH was 2.32 times higher in untreated patients, 1.54 times higher compared to warfarin, 4.38 times higher compared to dabigatran, and 1.27 times higher compared to apixaban, while it was similar to that of rivaroxaban [20]. Bauer et al. reported that, in patients undergoing TKA, the bleeding index was higher in the fondaparinux group than in the enoxaparin group, but no significant difference was found between the two groups with regard to major bleeding (retroperitoneal, gastrointestinal, intraocular, and intracranial bleeding) [21]. Similarly, Lassen et al. [22] evaluated patients that underwent THA and TKA, and they reported that no significant difference was seen between fondaparinux and enoxaparin with regard to major bleeding. In our study, in line with the literature, no major bleeding was observed in any patient following the administration of anticoagulant drugs.

In a meta-analysis study comparing LMWH and factor Xa inhibitors alone in patients undergoing THA, Lassen et al. [23] reported that the mortality rate was lower in patients receiving LMWH than in patients receiving factor Xa inhibitors. The authors also noted that the prevalence of clinically insignificant bleeding and minor bleeding was similar in patients undergoing THA and TKA. In our study, no mortality occurred in any patient.

Studies comparing LMWH and other drugs have found similar outcomes for these drugs with regard to efficacy and safety. Another meta-analysis study reported that although direct factor Xa inhibitors (rivaroxaban and apixaban) were found to be superior to enoxaparin in the prevention of DVT, no significant difference was found between these drugs in terms of complications including PE, major bleeding, and wound site infection and transfusion requirement. The authors also noted that direct factor Xa inhibitors have been preferred more commonly in post-TKA prophylaxis due to their ease of use [24].

Two recent randomized studies have compared the efficacy of aspirin and rivaroxaban in patients undergoing TKA and THA, and it was reported that aspirin and rivaroxaban were highly effective in the prevention of VTE [25, 26]. In a similar way, a randomized double-blind study has compared the efficacy of aspirin and low-dose rivaroxaban (10 or 20 mg), and it was reported that aspirin was better in preventing long-term VTE and in reducing relapse rates compared to rivaroxaban [26]. A previous meta-analysis compared the efficacy of enoxaparin (LMWH), placebo, and two main classes of anticoagulants (factor Xa inhibitors and direct thrombin inhibitors) for VTE prophylaxis in patients undergoing TKA and THA, and it was reported that LMWH was more effective than other three groups. On the contrary, no significant differences were found among the three drug classes with regard to the prevalence of PE and major bleeding [26]. In our study, LMWH and rivaroxaban showed similar efficacy in the prevention of DVT and also had similar rates of major and minor complications. Accordingly, we consider that rivaroxaban can be safely used for prophylaxis in patients undergoing TKA and THA.

As a result, the rivaroxaban is as effective as LMWH in the prevention of DVT. On the contrary, no significant difference was found between rivaroxaban and enoxaparin in terms of complications. It was also revealed that oral rivaroxaban provides greater ease of use compared to subcutaneous LMWH, which is an advantage of rivaroxaban over LMWH which may increase its preference in the prophylaxis of these surgeries.

## Figures and Tables

**Table 1:** Distribution of major complications

Major complications		Group 1 (46)	Group 2 (123)	Group 3 (45)	Total (214)

Bleeding causing a decrease in the amount of Hb more than 2 g/dl	10	5 (11.1%)	5 (4%)	0	10 (4.6%)

Bleeding causing more than two units of blood transfusion	16	0	10 (8.13%)	6 (13%)	16 (7.4%)

Bleeding causing treatment discontinuation or requiring reoperation for bleeding control	0	0	0	0	0

Symptomatic retroperitoneal, intracranial, intraocular, or intraspinal bleeding	0	0	0	0	0

Fatal bleeding	0	0	0	0	0

Total	26	5 (2.3%)	15 (7%)	6 (2.8%)	26 (23%)

**Table 2:** Distribution of minor complications.

Minor complications		Group 1 (46)	Group 2 (123)	Group 3 (45)	Total (214)

Ecchymosis larger than 25 square centimeters	45	10 (4.7%)	24 (11.2%)	11 (5.1%)	45 (21.0%)

Wound hematoma	0	0	0	0	0

Spontaneous nose or gum bleeding that lasts longer than five minutes	0	0	0	0	0

Spontaneous rectal bleeding	0	0	0	0	0

Spontaneous macroscopic hematuria	0	0	0	0	0

Total	45				45 (21.0%)

## Data Availability

No new data were used to support this study.
